# Neurobiophilia

**DOI:** 10.3390/brainsci16010085

**Published:** 2026-01-09

**Authors:** Mohamed Hesham Khalil, Koen Steemers

**Affiliations:** Neurocivitas Lab, Department of Architecture, University of Cambridge, Cambridge CB2 1PX, UK; kas11@cam.ac.uk

**Keywords:** neurosustainability, environmental neuroscience, climate change, nature, biophilic architecture and design, built environment, urbanisation

## Abstract

Despite unprecedented disconnection from nature and increased urbanisation, the brain still shows an affinity for nature. However, biophilia lacks a neuroscience foundation despite growing evidence of how the brain changes in response to the contrasting influences of urban and natural environments. To address this timely gap, this paper establishes Neurobiophilia through four objectives. First, it identifies seven neuro-needs (7NNs) and establishes their hierarchical order and interconnected outcomes. Second, it maps how natural environments fulfil each of the brain’s 7NNs. Third, it explores whether climate change is turning nature into a harmful environment for the brain, specifically with respect to temperature extremes. Fourth, it examines how built environments vary in their enrichment with respect to the 7NNs. This paper highlights critical environmental enrichment challenges in natural environments caused by climate change and in built environments. The novel Neurobiophilia framework established herein identifies these gaps and provides recommendations to achieve neurosustainability through environmental enrichment that sustains adaptive brain responses throughout the lifespan.

## 1. Introduction

At an unprecedented point in history, systematic reviews indicate that human connection to nature has declined globally over recent decades [1], as the built environment has continued to expand. Over two-thirds of the population is expected to live in cities by 2050 [2]; yet, regardless of biome or gender, people still have an affinity for natural elements in cities [3]. People benefit from visual exposure to natural elements in cities [3], and natural elements (e.g., tree cover density, sky visibility) in cities are associated with larger brain volumes [4,5,6]. This suggests the human brain has an affinity for nature, seeking environmental enrichment for its neurosustainability [7].

However, since the biophilia hypothesis was published around four decades ago [8,9], research on biophilia still lacks a neuroscience foundation [10], even though the term Neurobiophilia emerged a decade ago [11]. With environmental neuroscience evidence now accumulating, it is critical to address this gap as built environments expand and disconnection from nature intensifies, risking human brain health.

Thus, this paper establishes Neurobiophilia through four objectives. First, it identifies the brain’s neuro-needs and establishes their hierarchical and interrelated relationships. Second, it maps how natural environments nurture the human brain by fulfilling its neuro-needs. Third, it explores the potential impact of climate change on nature’s enrichment. Fourth, it examines how built environments vary in their enrichment.

## 2. The Brain’s Seven Neuro-Needs (7NNs)

**Figure 1:**
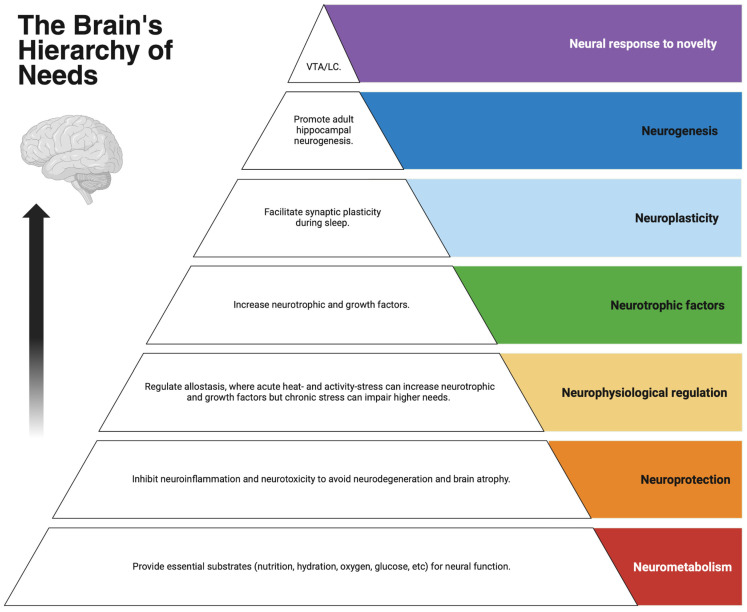
The brain’s hierarchy of needs.

**Figure 2:**
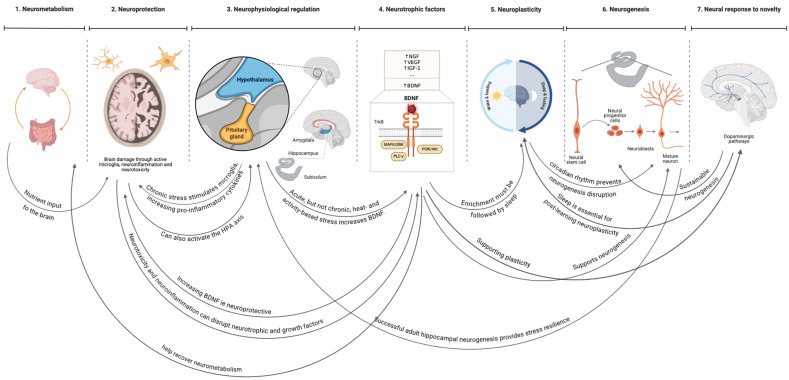
The interrelationships between the outcomes of the brain’s seven neuro-needs (7NN).

This section addresses the first objective by identifying the brain’s seven neuro-needs (7NNs) and establishing their hierarchical order and outcome interrelationships. Based on the review of evidence below, the 7NNs form a hierarchy (Figure 1): neurometabolism, neuroprotection, neurophysiological regulation, neurotrophic factors, neuroplasticity, neurogenesis, and neural responses to novelty. Whilst the needs are hierarchical, their outcomes are interrelated (Figure 2).

### 2.1. Neurometabolism

Neurometabolism is conceptually positioned at the foundational level, as the brain utilises essential energy sources to survive and sustain neural functions. At the very base level, the brain consumes about 20% of total body oxygen and utilises 25% of total body glucose [12,13,14]. Nutritional imbalances alter many properties of the neurovascular system [15], highlighting a feedback loop in neurometabolic function. Obesity can lead to severely impaired brain responses to post-ingestive nutrients [16]. Additionally, vitamin D deficiencies are also associated with changes in metabolic networks [17].

### 2.2. Neuroprotection

Neuroprotection is positioned second, since after providing the brain with the nutrients it needs to function, it becomes vital to protect it from high-risk factors, such as neuroinflammation and neurotoxicity, which can lead to brain damage and neurodegeneration. Neurotoxicity, the damage to the nervous system from disrupted function, can result from exposure to toxic environmental substances [18]. Pollutants are typical examples of neurotoxins [19,20,21,22] and can cause oxidative stress and inflammation [23], which in turn activate the hypothalamic–pituitary–adrenal (HPA) axis, leading to elevated stress hormones [24]. Severe heat stress (core body temperature reaching 42 °C in rats) can also lead to oxidative damage [25].

### 2.3. Neurophysiological Regulation

Neurophysiological regulation lies between neuroprotection and subsequent higher-level needs because it deals with stressors that are less harmful than neurotoxins; yet chronic stress may cause neuroinflammation [26,27], whereas acute, non-severe heat stress can be beneficial for higher-level needs, as explained subsequently. These nuances support the idea that neurophysiological regulation does not aim to prevent stress but rather supports recovery to gain benefit before harm occurs. When the allostatic response, the ability to adapt to stress, is overwhelmed, the costs of adaptation exceed the individual’s coping resources, resulting in allostatic overload [28,29,30]. Allostatic overload arises from chronic overactivation of the HPA axis and sympathetic–adrenal–medullary (SAM) axis, leading to physiological wear and tear [31]. Stress exposure also leads to amygdala hyperactivity [32], while the hippocampal subiculum plays a complex, stressor-specific role in regulating the HPA axis, primarily through inhibition [33]. Additional physiological markers can indicate whether stress exposure is chronic or acute [34]. These neurophysiological processes assist in determining whether the brain can recover after a brief period of exposure or is under chronic stress from external stimuli.

### 2.4. Neurotrophic Factors

Neurotrophic factors are included at the subsequent level, which is considered at a mid-level in the hierarchy of needs. Neurotrophic factors are a broad category that can encompass brain-derived neurotrophic factor (BDNF), nerve growth factor (NGF), vascular endothelial growth factor (VEGF), platelet-derived growth factor (PDGF), and insulin-like growth factor (IGF-1), among others.

The mid-level placement of neurotrophic factors is strategic for their input-output role. For instance, while BDNF benefits from acute heat stress to support the function of higher-level needs, as explained shortly, the outcome of BDNF secretion may also be therapeutic for dysfunctions at lower-level needs, such as BDNF-mediated support of neurometabolism and the neuroprotective role of BDNF. First, BDNF signalling enhances glucose metabolism by increasing glucose uptake, and acts on tropomyosin receptor kinase B (TrkB) to activate PI3K/Akt and mTOR pathways that stimulate the translation of glucose transporters to enhance cellular uptake of fuels. It regulates energy balance in both the central nervous system and peripheral metabolic organs, with roles in glucose homeostasis and insulin sensitivity [35,36]. Second, BDNF confers neuroprotection against mitochondrial dysfunction through anti-apoptotic effects, anti-oxidative actions through upregulation of antioxidant enzymes, and suppression of excessive autophagy [37]. Additionally, BDNF supports neurogenesis and plasticity, as explained in the following subsections.

BDNF levels increase through acute exposure to activity- or temperature-based stress [38]. Brief exposure to heat stress is associated with increased BDNF [39,40]. For instance, head-out immersion of males in hot water (42 °C) for 20 min increased BDNF [39]. Additionally, Kirby et al. [41] showed that BDNF increases by 90 pg/mL per 1 °C rise in ambient temperature (from 22 °C to 36 °C). Yet, severe heat stress (core body temperature reaching 42 °C in rats) can cause oxidative damage [25,42]. Physical activity can also increase BDNF and similar growth factors [43,44,45]. Both streams suggest that physical activity or temperature increases neurotrophic factors.

However, prolonged high-intensity physical activity or excessive heat stress can have adverse effects on BDNF levels. For instance, cortisol levels do not become counteractive as it increases through prolonged high-intensity training [46], while it was explained previously that severe heat stress (core body temperature reaching 42 °C in rats) can also lead to oxidative damage [25,42]. Chronic stress can also reduce hippocampal BDNF [47,48]. Understanding the thresholds becomes vital for avoiding harm.

Elevated neurotrophic factors can support the brain’s plasticity, a broad category that can be broken down into three primary, independent brain needs: neuroplasticity during sleep, neurogenesis, and neural responses to novelty. Those three needs constitute the highest-level brain needs in the given order.

### 2.5. Neuroplasticity

Neuroplasticity is the brain’s capacity to reorganise through synaptic strengthening, pruning, and the formation of new connections [49,50]. It occurs predominantly during sleep. The synaptic homeostasis hypothesis suggests that waking experience strengthens synapses (potentiation), while sleep enables selective downscaling and pruning, restoring synaptic strength to sustainable levels [51,52]. Thus, it is strategically positioned between the central brain need (neurotrophic factors) and higher-level needs (neurogenesis and neural responses to novelty), becoming a prerequisite for the latter two needs when they occur. Sleep remains essential after BDNF increases to fulfill its neuroprotective, pro-neurogenesis, and plasticity-promoting roles (as explained in previous and subsequent sections). Additionally, even dim light at night may reduce the expression of BDNF, adversely affecting neuroplasticity [53].

### 2.6. Neurogenesis: Adult Hippocampal Neurogenesis

Neurogenesis specifically refers to adult hippocampal neurogenesis in humans, as supported by growing research [54,55]. Cross-species analysis of adult hippocampal neurogenesis reveals human-specific gene expression alongside convergent biological processes [54], which facilitates understanding adult hippocampal neurogenesis by comparing environmental enrichment in human and non-human subjects.

To a great extent, through a physical activity-induced (e.g., running wheels) BDNF-dependent mechanism, the hippocampal dentate gyrus has the ability to generate new neurons in rodents [56]. For instance, BDNF binds to TrkB, which is largely expressed in hippocampal neurons. After binding, the BDNF-TrkB complex is then internalised into the neuron and supports proliferation, differentiation, maturation, and plasticity through signalling cascades that support neurogenesis [56]. The mediating role of BDNF between environmental enrichment and neurogenesis helps translate environmental enrichment from rodents to human environments, especially since BDNF-TrkB signalling is common between rodents and humans [57], similar to neurogenesis.

The comparison between rodents and humans is valid given that approximately 700 neurons are generated daily in each adult hemisphere’s hippocampal dentate gyrus, which the authors compared to non-human neurogenesis dynamics but highlighted differences in potential functional relevance [58]. Although this rate declines fourfold across the adult lifespan [58], another study found it to be stable with ageing [59]. Boldrini et al. [59] supported the persistence of adult hippocampal neurogenesis in humans reported by Spalding et al. [58] but showed that the decline with age was not found in their study.

Although a couple of early studies had reported undetectable neurogenesis in adults [60,61], subsequent evidence attributed this to methodological considerations [62]. Studies that followed continued to support the persistence of adult hippocampal neurogenesis in humans, even into the tenth decade of life [63,64,65].

Although neurogenesis in humans has been reported to potentially have different functional relevance from non-human subjects [58], contemporary research continues to explain the functional relevance, as neurogenesis drops in patients with Alzheimer’s disease (AD) risk and neuropsychiatric conditions [63,64,66]. Tobin et al. [63] reported persistent but variable adult hippocampal neurogenesis in aged AD patients (DCX+ mean 127,342 total per DG, Nestin+Sox2+ in 77.8%), yet with reduced proliferating DCX+PCNA+ neuroblasts compared to healthy subjects, suggesting early impairment in MCI/AD but potential resilience in some cases. Moreno-Jiménez et al. [64] reported a sharp, progressive decline in number and maturation in 45 AD patients aged 52–97 across Braak stages I–VI (modest reduction in early I–II, significant maturation failure with reduced PSA-NCAM/NeuN co-expression in III–IV, and near absence in V–VI).

Furthermore, an additional functional relevance of adult hippocampal neurogenesis that was studied in rodents but not yet confirmed in humans is the resilience to chronic stress that can be mediated by both increased hippocampal BDNF and enhanced neurogenesis [66,67,68,69,70].

### 2.7. Neural Responses to Novelty

Neural responses to novelty are positioned at the apex of brain needs because humans have a superior capacity to cognitively process novel information from diverse environmental sources, including aesthetics, activities, and recreation. There are different yet looped mechanisms through which novelty is processed.

First, the locus coeruleus (LC) is a small brainstem nucleus containing approximately 2000 neurons in rodents and roughly 30,000–50,000 neurons in humans that serves as the only source of norepinephrine (NE) for many forebrain regions. The LC becomes activated by prominent environmental signals such as novelty and stress, and LC-NE signalling enhances arousal and is critical for determining suitable affective and cognitive reactions to these stimuli [71]. LC responses are context-dependent and reflect cognitive significance rather than merely physical stimulus properties [72]. Importantly, these changes in LC responsiveness reflect specific information processing requirements rather than general arousal.

Second, substantia nigra/ventral tegmental area (SN/VTA), two closely located midbrain structures rich in dopamine-producing neurons, respond to stimulus novelty rather than other forms of salience such as rareness, emotional valence, or target detection [73]. The SN/VTA codes absolute rather than relative novelty magnitude, distinguishing it from adaptive coding mechanisms seen in reward processing. When familiar stimuli were presented in a context with novel stimuli, recognition memory was enhanced compared to presentation with very familiar stimuli.

Third, novelty exploration facilitates subsequent synaptic plasticity in a dopamine-dependent manner. To explain, dopamine-regulated synaptic plasticity supports the storage of unpredicted information in the hippocampal CA1 area [74]. The beneficial effects of novelty on memory are mediated by hippocampal dopamine, which comes from two different sources introduced earlier (the VTA or the LC) [75].

Collectively, since spatial novelty increases motivation and promotes learning [76], environments should provide periodic novelty to maintain the brain’s capacity for learning and memory formation and increase the neurotransmitters associated with exposure to novelty that may enhance mood as well, while static environments may lead to a decay in cognitive stimulation and inhibition of the release of key neurotransmitters.

## 3. How Nature Fulfils the Brain’s Neuro-Needs

**Figure 3:**
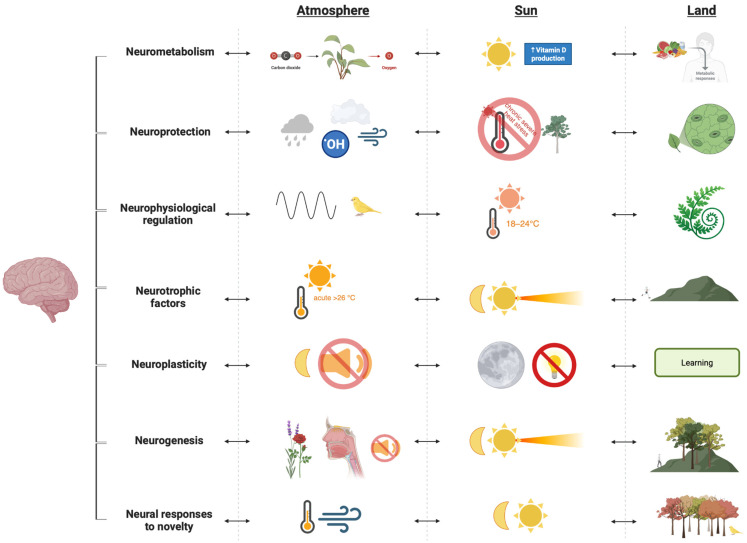
Neurobiophilia. Illustration of the brain–nature interactions.

This section addresses the second objective by examining how natural environments fulfil each of the brain’s seven neuro-needs (7NNs). The narrative synthesis is illustrated in Figure 3 and presented in Table 1.

Neurometabolism is well-supported in the natural environment. For instance, natural nutrients support brain function [77], while sunlight exposure is crucial for vitamin D production, which supports brain health [78,79,80]. The evidence not only supports the placement of neurometabolism at the base level of the hierarchy of needs but also shows how nature provides the brain with its basic need for survival and thriving.

Neuroprotection through the natural ecosystem is complex and does not seem to follow a linear relationship, at least under the contemporary definition of the natural environment, which has been impacted by several ecological changes. On the one hand, nature still has the capacity to eliminate chemical and gaseous neurotoxins and severe heat stress-induced pro-inflammatory responses. Firstly, nature manages the presence of chemical neurotoxins effectively through different mechanisms based on the size of the particulate matter (PM) via both wet deposition (where atmospheric pollutants are cleared by precipitation like rain, snow, or fog) and through dry deposition (on non-precipitation days, directly transferring airborne particles or through vegetation) [81,82,83,84,85]. Secondly, nature has mechanisms to eliminate various gaseous neurotoxins, including carbon monoxide (CO), carbon dioxide (CO_2_), nitrogen dioxide (NO_2_), volatile organic compounds (VOCs), and ground-level ozone (O_3_). CO is primarily removed through chemical reactions with hydroxyl radicals (•OH) in the air and biological consumption by soil microorganisms [86,87]. Last but not least, research shows that trees generally provide a cooling effect [88]. On the other hand, prolonged exposure to sunlight was found to be associated with reduced volumes of total brain, white matter, and grey matter [89], and exposure to severe heat stress (core body temperature reaching 42 °C in rats) may pose a neuroinflammatory risk to the brain [25]. The evidence supports the idea that nature nurtures the brain, but it also raises concerns about the source of the harmful effects of nature, as discussed in the subsequent section.

Neurophysiological regulation is well-explained by recent research. The term ‘environmental enrichment’ was applied to forests, finding that they are significantly associated with amygdala activity [90], which is responsible for emotional processing. Subsequent research showed that, after acute stress exposure, amygdala activity decreased following a 1 h walk in a natural environment (Grunewald Forest, Germany) [91]. The same experiment showed an adaptive response in the hippocampal subiculum, associated with stress responses [92]. Additionally, other studies support the idea that vegetation and natural soundscapes mitigate or recover from stress [93,94]. Evidence suggests that green natural environments can regulate neurophysiology; however, it is unclear whether other natural environments can achieve similar outcomes.

Neurotrophic factors can be significantly increased by distinct enrichment stimuli in nature, such as heat stress and affordances for physical activity. On the one hand, though we discussed earlier that exposure to severe heat stress can be pro-inflammatory [25], acute exposure to high temperatures can be a significant predictor of increased BDNF levels. For instance, seasonal variations are significantly associated with variability in serum and plasma BDNF levels [95,96], with BDNF levels higher in summer, suggesting that the natural ecosystem innately provides enrichment through heat stress. On the other hand, natural environments innately afford physical activity as a necessity of daily life, such as running, hunting, gathering, climbing, swimming, and walking on unpaved terrain. These activities, typically exceeding 3.5 METs [97], a threshold reported to increase BDNF levels through a gardening activity compared to a control group [45,98]. Interestingly, even 180 min of moderate-intensity walking (heat production of 200 W/m^2^) did not significantly increase BDNF levels in a temperate 18 °C environment compared to a hot 32 °C environment [99]. Collectively, the evidence suggests a central heat-based role in increasing BDNF, which can be supported by physical activity, both of which are provided by natural environments. To support this, physical activity is more common during the day in natural environments, and higher BDNF levels in summer are positively correlated with sunshine hours [95], suggesting a complementary role.

Neuroplasticity follows the previous brain needs at the brain’s hierarchy of needs. Previous evidence supports this positioning, as neuroplasticity builds on BDNF increases and requires sleep for neuroplastic changes to occur in the brain. Natural environments provide the light–dark cycles, whilst many brain disorders are correlated with circadian dysfunction [100].

Neurogenesis and how it changes in response to the environment are generally studied in non-human subjects such as mice and rats, whose adult hippocampal neurogenesis is comparable to that of humans [54,57], but comparisons between natural and laboratory environments suggest that nature provides similar benefits. First, environmental enrichment increases neurogenesis in rodents [101,102,103], primarily through navigational challenges supported by physical activity. For humans, it is unclear whether navigating a forest is associated with neurogenesis; however, evidence suggests that navigating forests and complex landscapes improves memory and maintains hippocampal volume, respectively. Weekly visits to forests correlate with improved cognitive performance in older adults [104], whilst other evidence suggests that navigating complex landscapes supports hippocampal volume in humans [105]. Second, olfactory enrichment, abundant in natural settings, may support hippocampal neurogenesis through odour novelty [106,107]. Third, bright light (10,000 lux) and maintenance of light–dark cycles enhance BDNF and neurogenesis [108,109,110,111]. Fourth, silence, unlike white noise, is associated with neurogenesis [112]. Therefore, it is possible to speculate that low neurogenesis in humans can be partially explained through the disconnection from nature’s environmental complexity and affordances for high metabolic equivalent (MET) activities.

Finally, neural responses to novelty are not yet supported by direct evidence due to a research gap. However, the circadian and seasonal changes inherent in natural environments suggest that future research should investigate these types of novelty.

## 4. The Potential Impact of Climate Change on Nature’s Enrichment

This section addresses the third objective by exploring whether climate change may compromise nature’s capacity to fulfil the brain’s neuro-needs. Climate change represents an urgent contemporary concern for both environmental and neurological health [113], as evidence suggests it may be diminishing nature’s enrichment.

The relationship between nature and neuroprotection is becoming increasingly complex under climate change conditions. As discussed in Section 3, prolonged exposure to sunlight (>2 h) is inversely associated with brain volume [89], and exposure to severe heat stress (core body temperature reaching 42 °C in rats) poses neuroinflammatory risks [25]. Climate change may explain or intensify these risks through multiple pathways.

First, temperature extremes can impair the brain’s resilience systems, either by inhibiting the increase in BDNF or causing neuroinflammation. Thereby, it can exacerbate or increase susceptibility to neurological disease [114]. A large-scale study of older adults found that for each 1 °C increase in high temperature, cognitive function scores decreased by 0.48 points, significantly greater than the 0.14-point decrease per 1 °C reduction in low temperature, demonstrating that heat poses a more substantial threat than cold to cognitive function [115].

Second, direct exposure of the head to solar heat radiation produces effects beyond ambient temperature alone. Prolonged exposure to simulated sunlight (approximately 1000 watt/m^2^) provokes core temperature elevation of 1 °C and significant impairments of cognitively dominated and motor task performances, with impairments emerging at considerably lower hyperthermia levels compared to experiments without radiant heating of the head [116]. Acute exposure did not affect any performance measures. This suggests that climate change-related increases in solar radiation intensity may pose additional neurotoxic risks beyond ambient temperature rises.

Third, sustained high body temperature exacerbates cognitive function and Alzheimer’s disease-related pathologies. Mice housed at a high ambient temperature (30 °C) for 13 months showed increased body temperature accompanied by memory impairment [117]. These findings suggest that chronic heat exposure from climate change may accelerate neurodegenerative processes.

Fourth, by 2050, more than 23% of the global population aged 69 and older will live in climates with acute heat exposure greater than the critical threshold of 37.5 °C, compared with 14% in 2020, an increase of 177–246 million older adults exposed to dangerous acute heat [118]. This demographic shift, combined with the vulnerability of older adults to heat-related cognitive decline, suggests a critical risk.

Fifth, climate change may be disrupting the natural seasonal variation in neurotrophic factors that traditionally provided cyclical enrichment. As discussed in Section 3, BDNF levels show seasonal variation, with higher levels in summer correlating positively with sunshine hours [95]. However, climate change may be altering this natural rhythm. The evidence suggests a hormetic relationship where moderate heat stress increases BDNF, whilst severe heat causes neuroinflammation, but climate change may be shifting baseline temperatures such that beneficial exposures become rarer whilst harmful exposures increase.

**Figure 4:**
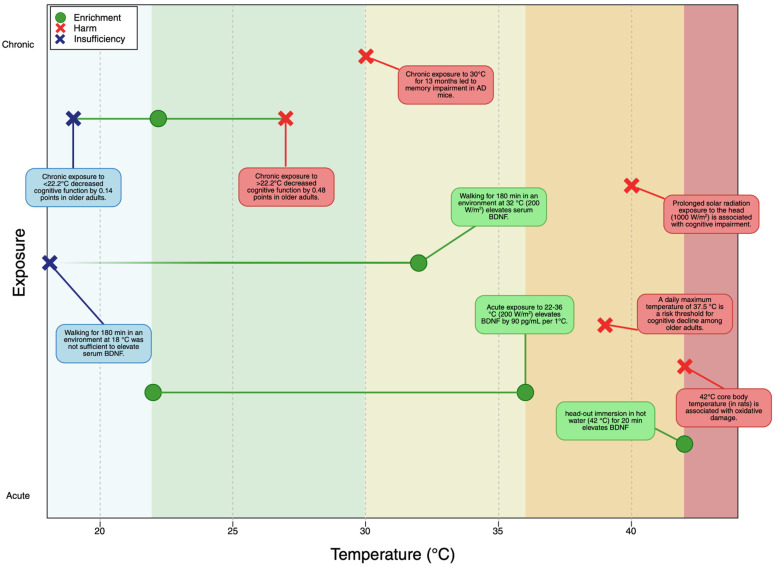
Heat exposure effects on brain function vary by temperature, duration, and age.

These climate change-related temperature effects present a paradox when compared with the beneficial heat stress discussed in Section 3. Section 3 established that acute exposure to moderate temperatures (22–36 °C) produces dose-dependent BDNF increases (90 pg/mL per 1 °C) [41], with even brief head-out water immersion at 42 °C (20 min) increasing BDNF [39], and seasonal variations showing higher BDNF in summer [95,96]. Additionally, 180 min of moderate-intensity walking produced greater BDNF increases at 32 °C compared to 18 °C [99]. However, the climate change literature reveals harmful effects from chronic moderate ambient heat (30 °C for 13 months) [117], acute extreme ambient heat (37.5 °C+) [118], prolonged solar radiation exposure to the head (1000 W/m^2^) [116], and severe heat stress reaching core body temperature of 42 °C causing oxidative damage in rats [42]. The distinction between beneficial and harmful heat appears to depend critically on exposure duration (20 min vs. 180 min vs. hours vs. months), whether heat affects core body temperature or remains peripheral, temperature intensity, and individual vulnerability. Notably, the same temperature (42 °C) can be beneficial with brief human head-out water immersion [39] yet harmful when it elevates core body temperature in rats [42], and the beneficial ambient temperature range (22–36 °C) overlaps with chronic exposure that causes harm (30 °C) [117]. Similarly, heat applied to the head can increase BDNF when delivered via water immersion [39] but impair cognition when delivered via prolonged solar radiation [116]. These overlapping ranges (Table 2 and Figure 4) and age differences highlight the complexity of heat–brain interactions.

The evidence reviewed suggests that climate change is creating a paradoxical situation where natural environments, traditionally viewed as enriching for the brain, may be developing harsh characteristics under climate change conditions.

## 5. Variability in Built Environment Enrichment

This section addresses the fourth objective by examining how built environments vary in their capacity to fulfil the brain’s seven neuro-needs (7NNs), contrasting their effects with the evidence presented in Section 3 on natural environments.

Built environments profoundly affect neurometabolism through more than one factor. Reduced walkability [119] and increased access to ultra-processed foods [120] directly compromise brain metabolism. These neurometabolic imbalances stem from insufficient opportunities for physical activity in the built environment. Walkability, in particular, affects multiple neuro-needs beyond neurometabolism, as demonstrated throughout this section.

Urbanisation compromises neuroprotection through multiple mechanisms. Macroenvironmental factors such as air, noise, and light pollution adversely affect the brain [121]. Research demonstrates an association between tree cover density in cities and larger brain volumes [4,5,122], with sky visibility explaining a significant portion of this effect [6]. Additionally, urban climate change produces serious adverse effects on brain-related health outcomes, further indicating inadequate neuroprotection [123].

Neurophysiological regulation represents one of the most widely studied yet complex neuro-needs. A critical unexplored problem concerns how the combination of sedentary behaviours and heightened stress leads to Type 2 allostatic overload [124], indicating that the challenge extends beyond merely eliminating stressors. Built environments, particularly city living, are associated with increased amygdala activity [125]. The contrast with natural environments is striking. Whilst forests show a significant association with reduced amygdala activity [90], urban green space demonstrates an insignificant association with the amygdala. The proximity of urban green space to urban stressors likely weakens this association, along with other factors such as tree cover density. Greater distance to the nearest major green space during pregnancy was associated with higher whole-brain fractional anisotropy [126], and impervious surfaces (grey space) are associated with increased amygdala-default mode network connectivity [127], indicating that built environments produce complex neurophysiological dysfunction. Furthermore, stress recovery shows reduced amygdala activity after a forest walk but no significant effect after a similar walk in an urban environment [91]. However, evidence remains insufficient to determine whether specific characteristics of built environments differentially affect amygdala activity following acute stress.

Built environments present multiple challenges for neurotrophic factors. First, urban infrastructure lacking affordances for physical activity exceeding 3 METs (e.g., walking, cycling) may increase the risk of low BDNF levels in populations. Second, cities with high pollution (e.g., near traffic roads) inhibit increases in BDNF levels even when physical activity opportunities are present [128], compared to environments with indoor air filtration. This further discourages outdoor physical activity. Third, whilst stair use increases BDNF [129], it cannot completely compensate for outdoor experiences due to the need for structured or prolonged stair use. Fourth, indoor environments can provide solutions to increase BDNF (e.g., saunas, hot tubs) [39,130], delivering acute heat stress without the chronic effect. The built environment thus presents both outdoor and indoor challenges affecting BDNF levels, calling for investigation to enrich both scales of the built environment.

Built environments threaten neuroplasticity primarily through disruption of the light–dark cycles essential for optimal brain function. As established in Section 3, neuroplasticity depends critically on proper circadian rhythms and sleep architecture. However, multiple features of modern built environments systematically undermine these requirements. First, noise pollution from traffic, construction, and late-night commercial activities can severely disrupt sleep when buildings lack appropriate acoustic design. Extended commercial operating hours into late evening can further increase the likelihood that populations fail to follow proper circadian rhythms. For instance, shift workers and individuals with irregular sleep schedules show reduced hippocampal volume and impaired memory consolidation [131], a line of evidence that supports the previous sections linking circadian disruption to compromised neuroplasticity. Second, indoor artificial light, a modern built environment feature, represents an equally serious threat. Indoor environments enable active evening lifestyles through bright artificial lighting, disrupting the natural light–dark cycles required for neuroplasticity. Light exposure during evening hours suppresses melatonin production and delays circadian phase [132,133]. Shifted light–dark cycles disrupt genes involved in stress, neuroplasticity, and motivation in mice [134]. These cumulative factors in built environments create a circadian disruption cascade that fundamentally impairs the neuroplastic processes dependent on proper sleep architecture.

Neurogenesis in populations may vary dramatically depending on four factors: urban infrastructure affordances for physical activity, architectural affordances for physical activity, geospatial complexity, and architectural/indoor complexity. Beyond the physical activity factors addressed earlier in this section, geospatial complexity may predict adult hippocampal neurogenesis in humans. High geospatial complexity is associated with larger hippocampal and other brain regions, as well as lower Alzheimer’s disease risk [135]. Given that neurogenesis declines in patients with Alzheimer’s disease [62,63], urban environments can be evaluated based on their geospatial complexity to assess neurogenic potential.

Neural responses to novelty in built environments present a complex paradox. Cities potentially provide higher novelty exposure than natural environments through diverse architectural forms, changing streetscapes, varied social encounters, and dynamic commercial environments. However, whether this constitutes understimulation, optimal stimulation, or overstimulation remains unclear and likely varies across individuals and contexts. The nature of urban novelty differs fundamentally from natural novelty. Whilst natural environments provide novelty through seasonal changes, weather variations, and encounters with diverse flora and fauna, urban novelty tends toward rapid, frequent, and often unpredictable stimuli. This difference may have important neurological implications. Excessive novelty, particularly when combined with stressors, may lead to cognitive overload and habituation rather than adaptive neural responses. Additionally, accessibility to novelty is often restricted by socioeconomic, architectural, and temporal barriers, which creates substantial variation in neurogenic enrichment across urban populations.

## 6. Future Research

The human brain is no longer shaped by nature alone. Four converging trends underscore this shift: disconnection from nature, climate change impacts on nature’s enrichment capacity, urban population growth (68% of the world population expected to inhabit cities by 2050), and extensive indoor living (approximately 90% of time spent inside buildings) [136,137,138]. Table 3 synthesises the findings by contrasting enrichment across natural, urban, and indoor environments for each of the seven neuro-needs (7NNs). This comparison reveals substantial gaps requiring targeted investigation.

Future research should employ targeted methodologies to address these gaps. Neuroprotection can be assessed via magnetic resonance imaging (MRI) to quantify structural brain changes across environments. Neurophysiological regulation requires multiple approaches: MRI for acute changes in amygdala activity and subiculum volume, or electroencephalography (EEG). Neurotrophic factors responding to brief, non-severe heat stress or physical activity can be measured through changes in brain-derived neurotrophic factor (BDNF) levels in blood serum and plasma, though salivary methods are not yet reliable [139]. Neuroplasticity can be tested by measuring changes in brain activity and structure. Neurogenesis and its associated outcomes, such as Alzheimer’s disease risk [62,63], neuropsychiatric conditions [66], and pattern separation ability [140,141], require longitudinal study designs. Functional magnetic resonance imaging (fMRI) can help measure activity in deep brain structures like the VTA and LC in humans [142].

Collectively, these investigations would determine whether contemporary environments, natural under climate change or built under current design paradigms, adequately support the brain’s seven neuro-needs, and establish evidence-based design principles for creating environments that support neurosustainability.

## 7. Conclusions

At a critical juncture when most of humanity inhabits cities and spends 90% of their time indoors, whilst the remaining time spent outdoors is increasingly compromised by air pollution and climate change, the Neurobiophilia framework provides the neuroscience foundation that biophilia has lacked for four decades. Environmental enrichment faces unprecedented threats: climate change transforms beneficial heat exposure into harmful extremes, air and noise pollution concentrate in urban areas where most people live, and extensive indoor living deprives populations of an increase in neurotropic factors. Yet, these challenges are addressable through evidence-based design. Achieving neurosustainability demands immediate translation of environmental neuroscience into actionable principles that fulfil the brain’s seven neuro-needs, ensuring adaptive neural responses across natural, urban, and indoor environments throughout the lifespan.

## Figures and Tables

**Table 1:** Neurobiophilia. A summary of the brain–nature interactions.

7NNs	Nature’s Enrichment		
	Atmosphere	Sun	Land
Neurometabolism	Provision of oxygen for neural function	Sunlight enabling vitamin D synthesis for brain function	Nutrients water and diverse food sources
Neuroprotection	Filter pollutants through wet/dry deposition	Non-chronic heat exposure (before climate change)	Plant stomata and soil microorganisms
Neurophysiological regulation	Natural soundscapes facilitate stress recovery	Natural daylight and temperature 18–24 °C	Vegetation density and moderate fractal dimensions
Neurotrophic factors	Brief exposure to heat stress > 26 °C	Bright light > 10,000 lux, and dark/light cycles	Affordances for activity > 3.5 metabolic equivalents
Neuroplasticity	Lowest noise levels at night	Light–dark cycles facilitate sleep-dependent plasticity	Learning through the environment
Neurogenesis	Natural scents and the absence of sounds at night	Bright light > 10,000 lux, and dark/light cycles	Activity > 3.5 METs and complex navigation
Neural responses to novelty	Atmospheric and temperature changes	Day and night variations and duration dynamics	Seasonal changes and duration dynamics

**Table 2:** Contrasting beneficial and harmful heat exposure effects on the brain.

Exposure Type	Temperature	Duration	Outcome
Head-out water immersion	42 °C	20 min	↑ BDNF (beneficial) [39]
Ambient heat (healthy adults)	22–36 °C	Acute (unspecified)	↑ BDNF 90 pg/mL per 1 °C [41]
Walking + heat (200 W/m^2^)	32 °C vs. 18 °C	180 min	Greater BDNF at 32 °C [99]
Core body temperature	42 °C	N/A	Oxidative damage [25,42]
Solar radiation (head)	1000 W/m^2^	Hours (unspecified)	Cognitive impairment [116]
Ambient heat (AD mice)	30 °C	13 months	Memory impairment [117]
Population heat exposure (older adults) *	Deviation from 22.2 °C	Chronic/seasonal	Cognitive decline per 1 °C increase than per 1 °C decrease [115]
Population heat exposure (older adults) *	>37.5 °C	Daily maximum	Cognitive decline risk [118]

↑ = Increase. * temperature, duration and effect on the brain are respective to older populations.

**Table 3:** Comparison of environmental enrichment in natural, urban, and indoor environments.

7NNs	Natural Environment	Urban Environment	Indoor Environment
Neurometabolism	Nutrients from diverse food sources; vitamin D from sunlight; physical activity through terrain navigation.	Reduced walkability; increased access to ultra-processed foods; insufficient physical activity opportunities.	Sedentary behaviour; lack of natural light for vitamin D synthesis; controlled access to food quality varies by setting.
Neuroprotection	Elimination of neurotoxins via wet/dry deposition, but prolonged sun exposure reduces brain volume.	Air, noise, and light pollution; tree cover density and sky visibility associated with larger brain volumes.	Air filtration systems can protect from outdoor pollution.
Neurophysiological regulation	Forests reduce amygdala activity; stress recovery after brief nature walks.	City living increases amygdala activity, and it fails to reduce its activity after acute stress.	Sedentary behaviour and stress lead to Type 2 allostatic overload.
Neurotrophic factors	Heat stress increases BDNF (90 pg/mL per 1 °C, 22–36 °C) and supports physical activity-induced BDNF; seasonal BDNF variation can be impacted by climate change.	Infrastructure lacking affordances for physical activity > 3 METs; pollution inhibits BDNF increases even with physical activity opportunities.	Controlled solutions available: saunas, hot tubs provide acute heat stress; stair use can increase BDNF, but requires prolonged use.
Neuroplasticity	Natural light–dark cycles support sleep and circadian rhythms essential for neuroplasticity.	Noise pollution disrupts sleep; extended commercial hours prevent proper sleep patterns.	Artificial lighting disrupts natural light-dark cycles, increasing evening lifestyles that delay circadian phase.
Neurogenesis	Navigational challenges; olfactory enrichment; bright natural light; silence opportunities.	Variable effects: high geospatial complexity → larger hippocampus, lower AD risk; urban infrastructure affordances for physical activity are critical.	Architectural affordances for physical activity (e.g., stair design); architectural/indoor complexity may provide navigational challenges.
Neural responses to novelty	Seasonal changes; weather variations; diverse flora/fauna encounters; gradual and cyclical novelty; circadian and seasonal variations urge investigation.	Potentially higher novelty through architectural diversity, social encounters; rapid and frequent stimuli; unclear if understimulation, optimal stimulation, or overstimulation; accessibility often restricted.	Monotonous environments with limited spatial variation; repetitive daily routines; insufficient novelty in the built environment.

## Data Availability

The original contributions presented in this study are included in the article.
